# Biodegradation of Quinoline by a Newly Isolated Salt-Tolerating Bacterium *Rhodococcus gordoniae* Strain JH145

**DOI:** 10.3390/microorganisms10040797

**Published:** 2022-04-09

**Authors:** Yinhu Jiang, Fuyin Zhang, Siqiong Xu, Pan Yang, Xiao Wang, Xuan Zhang, Qing Hong, Jiguo Qiu, Cuiwei Chu, Jian He

**Affiliations:** 1 Key Laboratory of Agricultural Environmental Microbiology of Ministry of Agriculture, College of Life Sciences, Nanjing Agricultural University, Nanjing 210095, China; 2020116056@stu.njau.edu.cn (Y.J.); 2020116043@stu.njau.edu.cn (F.Z.); 2020216018@njau.edu.cn (S.X.); 2021116060@stu.njau.edu.cn (X.W.); 2021216024@stu.njau.edu.cn (X.Z.); hongqing@njau.edu.cn (Q.H.); qiujiguo@njau.edu.cn (J.Q.); 2 College of Life Sciences and Agronomy, Zhoukou Normal University, Zhoukou 466000, China; 20182012@zknu.edu.cn

**Keywords:** quinoline, biodegradation, salt-tolerance, *Rhodococcus*, JH145, new degradation pathway

## Abstract

Quinoline is a typical nitrogen-heterocyclic compound with high toxicity and carcinogenicity which exists ubiquitously in industrial wastewater. In this study, a new quinoline-degrading bacterial strain *Rhodococcus* sp. JH145 was isolated from oil-contaminated soil. Strain JH145 could grow with quinoline as the sole carbon source. The optimum growth temperature, pH, and salt concentration were 30 °C, 8.0, and 1%, respectively. 100 mg/L quinoline could be completely removed within 28 h. Particularly, strain JH145 showed excellent quinoline biodegradation ability under a high-salt concentration of 7.5%. Two different quinoline degradation pathways, a typical 8-hydroxycoumarin pathway, and a unique anthranilate pathway were proposed based on the intermediates identified by liquid chromatography–time of flight mass spectrometry. Our present results provided new candidates for industrial application in quinoline-contaminated wastewater treatment even under high-salt conditions.

## 1. Introduction

Quinoline and its derivatives are common nitro-heterocyclic compounds in industry, which are refractory and widely distributed in industrial wastewater, such as coking wastewater and pulping wastewater [1,2,3,4,5,6]. Compared with other polycyclic aromatic hydrocarbons, quinoline more easily enters groundwater and soil due to its unique heterocyclic structure. Quinoline was identified as a toxic pollutant due to its carcinogenic and mutagenic properties. At present, the removal of quinoline pollutants from wastewater is of great interest.

Generally, quinoline has been removed by physical, chemical, or biological methods [7]. Among these methods, the physical methods have generally included adsorption, dissolution, and filtration [8]. A variety of chemical technologies have been applied for removing wastewater containing quinoline, such as wet oxidation [9,10] and ultraviolet (UV) photolysis [11,12,13]. Based on the present status of quinoline wastewater treatment, several methods have usually be combined to improve the removal efficiency. However, compared with physical or chemical methods, biological treatment has become a promising technology for removing various refractory pollutants, including quinoline, due to its merits that include less chemical agents, equipment, and secondary pollution [14,15,16,17].

Microorganisms play a major role in the biodegradation of quinoline in the environment. Several quinoline-degrading strains under aerobic or anaerobic conditions have been reported in recent years, such as *Burkholderia* sp. [18,19], *Bacillus* sp. [20], *Brevundimonas* sp. [21], *Rhodococcus rhodochrous* [22], *Comamonas* sp. [23,24], and *Pseudomonas* sp. [25,26]. However, most quinoline-degrading bacteria has shown relatively low adaptability and low degradation efficiency. Furthermore, many industrial wastewaters contain high concentrations of salt components, thus, the salt tolerance of bacterial strains plays an important role in wastewater treatment [6]. Nevertheless, high salt-tolerating quinoline-degrading bacteria have been investigated poorly in previous studies.

In this study, a new quinoline-degrading bacterial strain JH145 with high efficiency and halotolerance was isolated. The characteristics of quinoline degradation by strain JH145 were examined under different cultural conditions. The degrading intermediates were investigated and the pathways were proposed. The present findings will improve our understanding of quinoline biodegradation in a different environment.

## 2. Materials and Methods

### 2.1. Chemicals and Media

Quinoline (96% purity) and 2-hydroxyquinoline (97% purity) were purchased from Macklin Biochemical (Shanghai, China). These chemicals were dissolved in deionized water before use. Methanol used in high-performance liquid chromatography (HPLC) was purchased from Merck KGaA (Darmstadt, Germany). Luria-Bertani (LB) medium used to enrich bacteria contained: tryptone (10.0 g/L), yeast extract (5.0 g/L), and NaCl (10.0 g/L), at pH 7.0. The mineral salt medium (MSM) used for the bacterial growth and bacterial isolation contained (NH4)_2_SO_4_ (1.0 g/L), KH_2_PO_4_·2H_2_O (0.5 g/L), MgSO_4_·7H_2_O (0.2 g/L), K_2_HPO_4_·3H_2_O (1.5 g/L), NaCl (1.0 g/L), at pH 7.0. The corresponding solid media were prepared by adding 1.8% agar powder. All the above culture media needed to be sterilized in an autoclave (121 °C, 25 min) before being used.

### 2.2. Enrichment and Isolation of Bacteria

Oil-contaminated soils were collected to isolate quinoline-degrading bacteria. First, the oil-contaminated soil (35°21′–37°31′ N, 107°41′–110°31′ E) from an oil field was added into the sterile MSM (250 mL) containing 100 mg/L quinoline and then incubated in a shaker at 30 ℃ and 180 rpm. After cultivation for 7 days, the cell suspension (10 mL) was transferred and inoculated into another fresh MSM (250 mL) with 100 mg/L quinoline. The above cultures were repeated 5 times, then serially diluted and coated in MSM plates with 100 mg/L quinoline. The plates were incubated at 30 °C for several days. After visible colonies were formed on plates, individual colonies with different morphology were selected and strewn on MSM plates with 100 mg/L quinoline. The removal capacity of quinoline by the purified strain was verified. Finally, a strain of *Rhodococcus* sp. JH145 with high quinoline-degrading efficiency was selected for further study.

### 2.3. Identification of Strain JH145

The high-salt precipitation method was used to extract the genomic DNA of strain JH145. The 16S rRNA gene was amplified using the universal primers 27F and 1492R [27]. The amplified PCR product was purified and recovered with the gel recovery kit of Nanjing Novoz Biotech (Nanjing, China) Co., Ltd. The qualified product was further connected to pMD19-T and then was introduced into E. coli DH5α. Positive clones were screened on Luria-Bertani (LB) plates containing 100 mg/L ampicillin and then sequenced by Sangon Biotech Co., Ltd. (Shanghai, China). The obtained sequence was compared with those of the related microorganisms by BLAST and then identified as *Rhodococcus* sp. Finally, the phylogenetic tree of this strain was constructed with MEGA6 by using the neighbor-joining method.

### 2.4. Characteristics of Quinoline Biodegradation by Strain JH145

The strain JH145 was cultured in LB, collected, and washed twice with MSM. The cells were suspended at OD600 of 2.0. Then the degradation ability of strain under various environmental conditions was investigated in 250 mL Erlenmeyer flasks. First, the fresh bacterial solution was added in 100 mL MSM containing 100 mg/L quinoline with 1% inoculum. The biomass was measured by OD600nm and degradation of quinoline was monitored at the same time by sampling regularly.

Next, tests on the effects of temperature were examined at five levels 15, 20, 30, 37, and 45 °C, at pH 7.0. Then these were followed by tests on the effects of pH at six levels from pH 5.0 to 10.0 at the identified optimal temperature. Finally, at the optimal temperature and pH, the effects of salt concentration levels (1% to 10% [*w*/*v*]) on quinoline degradation were tested. All samples were tested in triplicate.

### 2.5. Detection of Quinoline Metabolites and Analysis Method

Samples at regular intervals of 4 h within 2 days were taken out from Erlenmeyer flasks, centrifuged for 5 min at 12,000 rpm, and then the supernatant was gathered. The concentrations of quinoline and its intermediates in the supernatant were analyzed by high-performance liquid chromatography (HPLC), using a Syncronis C18 column (4.6 μm × 250 mm × 5 μm). The methanol solution with (40:60, *v/v*) ultrapure water containing 1% formic acid was used as mobile phase at the flow rate of 1.0 mL min-1 by 20 μL injection volume. The UV detector was set at 275 nm.

To detect the metabolism intermediates of quinoline, the supernatant from the Erlenmeyer flask was lyophilized, dissolved by methanol, and then analyzed by liquid chromatography–time of flight mass spectrometry (LC–TOF/MS). By analyzing the positive and negative ion patterns, the metabolism intermediates of quinoline were determined by comparing literature and the standards [28].

## 3. Results and Discussion

### 3.1. Identification of Quinoline Degrading Bacteria

Quinoline was used as the sole carbon source for the enrichment culture. The fifth-generation enrichment solutions were applied for gradient dilution. Through continuous streaking and purification on the MSM plate with 100 mg/L quinoline as the sole carbon source, a single strain named JH145 was finally obtained. Strain JH145 is a Gram-positive bacteria, and its colony appears red, circular, slightly convex, and surface drying. Strain JH145 could degrade tyrosine but not casein, hypoxanthine, or xanthine. It hydrolysed aesculin weakly and isindole- and urease-negative. Meanwhile, strain JH145 was resistant to streptomycin and ampicillin, but susceptible to kanamycin and gentamicin. The strain was also positive for catalase and could grow in the presence of lysozyme, without production of H_2_S. At the same time, the phenotype and colony characteristics of this strain were very similar to those of *Rhodococcus* [20].

**Figure 1:**
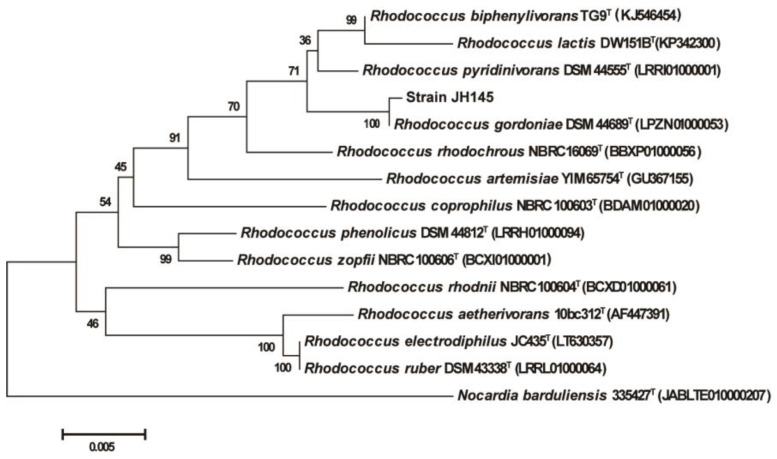
Phylogenetic tree constructed from the 16S rRNA sequence of strain JH145 and some other strains by the neighbor-joining method.

The BLAST search of the 16S rRNA gene sequence indicated that strain JH145 was closely related to the species in the genus of *Rhodococcus*, and also exhibited the highest similarity to *Rhodococcus gordoniae* (99.64%) and *Rhodococcus pyridinivorans* (98.79%) (Figure 1). Based on phenotypic and 16S rRNA gene phylogenetic analysis, the strain JH145 was identified as *Rhodococcus* sp. According to previous research reports, some strains in *Rhodococcus* can degrade quinoline under aerobic culture conditions, such as *Rhodococcus pyridinivorans* [29], *Rhodococcus* sp. Bl [30], and *Rhodococcus rhodochrous* [22]. The quinoline-degrading bacterial strains were diverse, and strain JH145 is the first strain of *Rhodococcus gordoniae*. reported to degrade quinoline.

### 3.2. Growth Status and Degradation Characteristics of Strain JH145

#### 3.2.1. Study on the Growth Profile of Quinoline-Degrading Strain JH145

**Figure 2:**
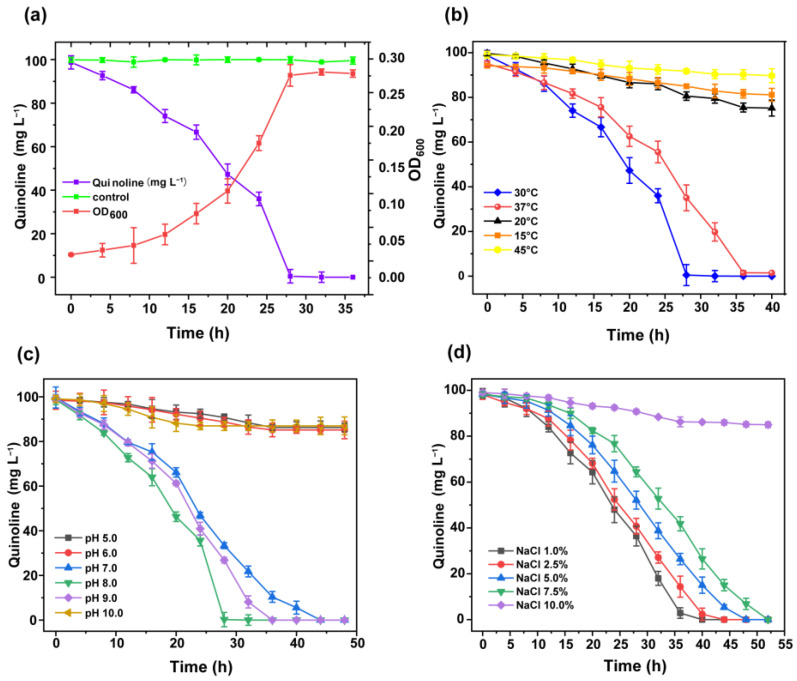
Effect of JH145 on quinoline degradation under different conditions (**a**) The cell growth and degradation characteristics of strain JH145 with quinoline as the sole carbon source. (**b**) Effects of temperature on the quinoline biodegradation of strain JH145. (**c**) Effects of initial pH on the quinoline biodegradation of strain JH145. (**d**) Effects of NaCl on the quinoline biodegradation of strain JH145.

The bacterial suspension with 1% inoculum was added into the 100 mL sterile MSM with 100 mg/L quinoline, at pH 7.0, and then the flask was cultured in a shaker at 30 °C and 180 rpm. Figure 2a shows that 100 mg/L quinoline was degraded completely within 30 h. Meanwhile, the bacterial cells were grown up to OD600nm of 0.27 ± 0.02. The growth rate of strain JH145 was slow in the first 14 h due to the adaptation period to this medium. After 14 h, the growth rate of the strain increased significantly, indicating that strain JH145 entered the logarithmic growth phase and removed quinoline efficiently, and utilized it as the carbon source for cell growth.

#### 3.2.2. Study on the Degradability of Quinoline by Temperature

Temperature is a significant factor for quinoline degradation in the environment. As shown in Figure 2b, 100 mg/L quinoline was degraded completely by strain JH145 within 36 h at 30 °C and 37 °C, respectively. The quinoline degradation at 37 °C was a little slower than that at 30 °C. However, quinoline was hardly degraded by strain JH145 at 15 ℃, 20 °C, and 45 °C. The degradation rates were less than 20% of 100 mg/L quinoline. The order of quinoline degradation rates was as follows: 30 °C > 37 °C >> 20 °C > 45 °C > 15 °C. It might be due to the fact that strain JH145 is a mesophilic bacteria and the enzymes involved in quinoline biodegradation were temperature-sensitive.

#### 3.2.3. Study on the Degradability of Quinoline by pH

The MSM with different pH values of 5.0, 6.0, 7.0, 8.0, 9.0. and 10.0 were adjusted by a 50 mM NaH_2_PO_4_-Na_2_HPO_4_ buffer system. The temperature used for degradation was 30 °C as investigated above. Figure 2c illustrates that 100 mg/L quinoline was degraded completely by strain JH145 at the initial pH of 7.0, 8.0, and 9.0. The optimum pH value for quinoline degradation was 8.0. The order of quinoline degradation rates was as follows: pH 7.0 > pH 8.0 > pH 9.0. Therefore, the strain JH145 could degrade quinoline well in neutral and light alkaline environments. In contrast, strain JH145 never degraded the added quinoline at the initial pH of 5.0, 6.0, and 10.0, indicating that extreme acidic or alkaline conditions were harmful to strain JH145. Since the actual growth environment of the bacteria was mostly a neutral environment, the following experimental conditions were set at pH 7.0.

#### 3.2.4. Study on Salt Tolerance of Strain JH145

Figure 2d shows the quinoline removal by strain JH145 at different initial concentrations (1.0%, 2.5%, 5.0%, 7.5%, and 10%) of NaCl. 100 mg/L quinoline was degraded well with 1.0~7.5% NaCl within 50 h by strain JH145. With the increase of NaCl concentration from 1.0% to 7.5%, the removal rate of quinoline slightly decreased gradually. Nevertheless, only approximately 15% of the initial quinoline was consumed at 50 h when NaCl reached 10.0%, probably due to the inhibition effects of NaCl concentration on biodegradability.

In the current wastewater treatment by bacteria, high salt concentration conditions are a common problem [30]. Bacteria does not degrade pollutions in those high salt concentration conditions probably due to the high osmotic pressure of salt. Our present results showed that strain JH145 could remove quinoline efficiently under NaCl concentration of up to 7.5%, indicating the superiority of strain JH145 in industrial treatment high-salt concentration quinoline wastewater.

### 3.3. Identification of Metabolites and Proposal of Quinoline Aerobic Catabolism Pathway

**Figure 3:**
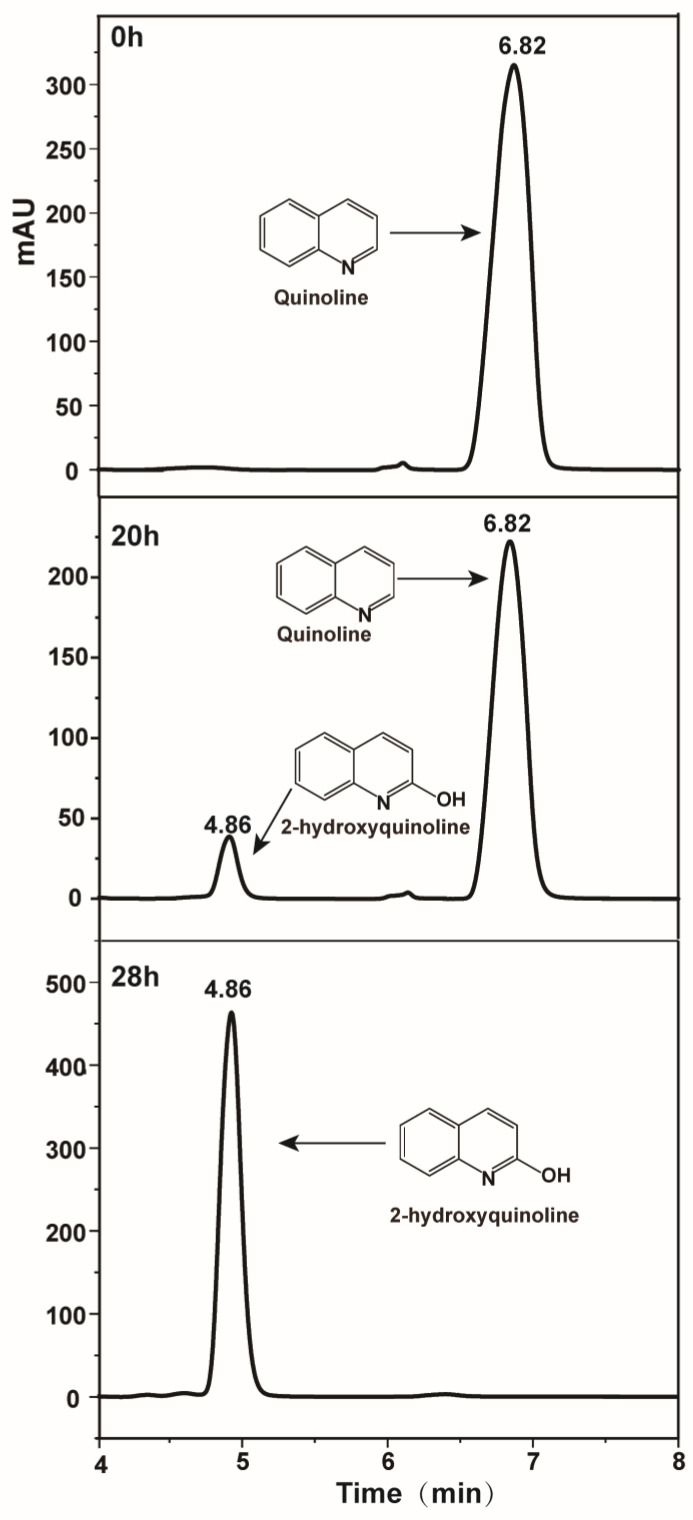
HPLC chromatograms of quinoline and 2-hydroxyquinoline.

**Figure 4:**
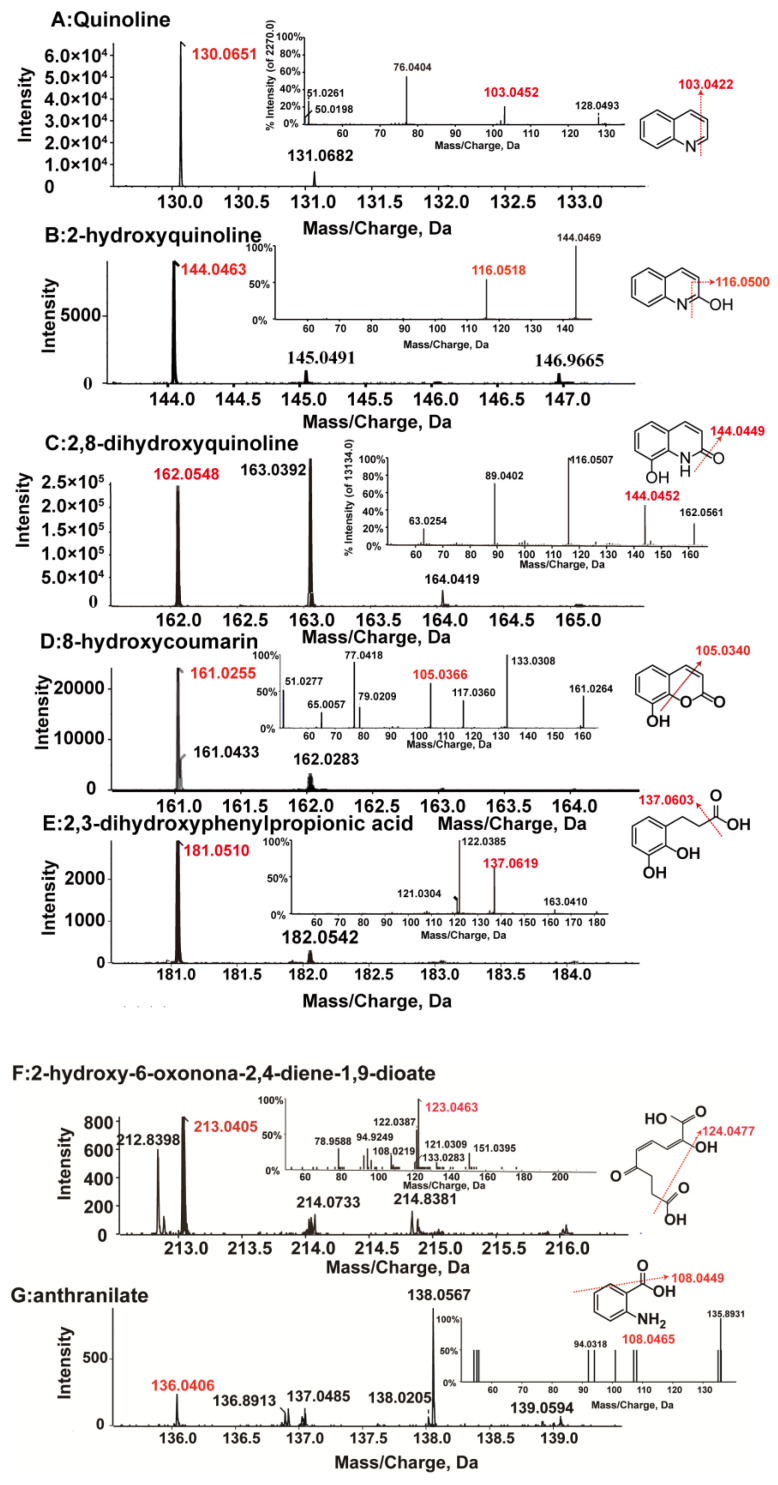
HPLC–MS mass spectrograms and proposed structures of quinoline and its metabolites.

HPLC was used to detect the possible catabolic intermediates during quinoline degradation. As shown in Figure 3, a new peak around 4.86 min appeared when quinoline (6.82 min) degraded and finally disappeared. LC-MS results of this new peak showed a molecular ion peak at m/z 144.0463 [M-H] and fragment ion peak at 116.0518 [M-H], indicating the adding of the oxygen atom. Generally, the first step of the aerobic degradation of quinoline was 2-hydroxyquinoline as shown in Figure 4B [23,29], reported in almost all quinoline biodegradation pathways. HPLC results showed that the retention time of the new peak was equal to that of the commercial 2-hydroxyquinoline standard. Thus, the new peak was identified as 2-hydroxyquinoline.

**Figure 5:**
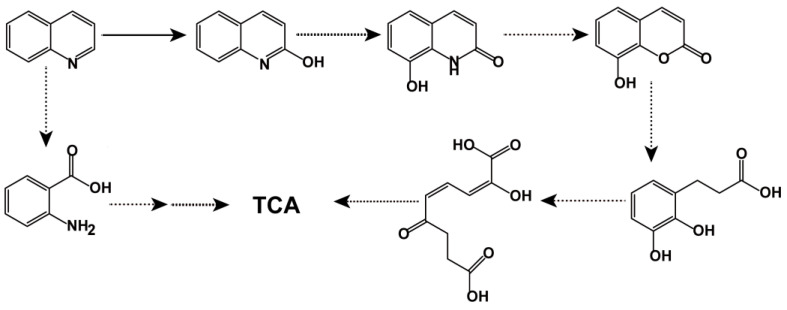
The proposed quinoline biodegradation pathway of strain JH145.

Although only one visible peak emerged in HPLC analyses, several trace amount intermediates were detected by HPLC–MS. As shown in Figure 4, five more metabolites were detected. According to their molecular and fragment ion peaks, these intermediates were proposed as 2-hydroxyquinoline (Figure 4B), 2,8-dihydroxyquinoline (Figure 4C), 8-hydroxycoumarin (Figure 4D), 2,3-dihydroxyphenylpropionic acids (Figure 4E), 2-hydroxy-6-oxonona-2,4-diene-1,9-dioate (Figure 4F), and anthranilate (Figure 4G). Based on these intermediates, two different quinoline biodegradation pathways in strain JH145 were proposed (Figure 5). In the first path, 2-hydroxyquinoline was converted to 2,8-dihydroxyquinoline. Afterward, 2,8-dihydroxyquinoline was converted to 8-hydroxycoumarin and accompanied by NH_4_^+^, which underwent ring-opening to form 2,3-dihydroxyphenylpropionic acids. Thereafter, the catabolic compound was meta-cleaved to yield 2-hydroxy-6-oxonona-2,4-diene-1,9-dioate, followed by downstream metabolism ending up in the citrate cycle (TCA). In another pathway, the quinoline cleaved the ring to form an anthranilate. As no other intermediates were observed in the experiments, subsequent metabolites of anthranilate were unclear.

As shown in previous studies, two common quinoline degradation pathways named the 5,6-dihydroxy-2(1H) quinolinone pathway and the 8-hydroxycoumarin pathway, exist in quinoline-degrading microorganisms [31,32]. Bacteria usually employ one quinoline degradation pathway, for example, *Comamonas* with the 8-hydroxycoumarin pathway [23]. Recently, quinoline was found to be removed by two pathways in one strain, such as *Rhodococcus* [28] and *Pseudomonas putida* [33]. In this study, we found that the 8-hydroxycoumarin pathway and, interestingly, a unique anthranilic acid pathway were used by *Rhodococcus* sp. JH145. This unique anthranilic acid degradation pathway did not appear in Gram-positive bacteria but was reported in a Gram-negative bacterium *Comamonas* sp. recently [34]. Considering the significant differences of Gram-positive and Gram-negative bacteria, more studies of *Rhodococcus* sp. JH145 are expected in our future research.

## 4. Conclusions

In this study, a new quinoline-degrading strain JH145 was isolated from oil-contaminated soil. The strain was identified as *Rhodococcus* sp. based on its morphology and 16S rRNA gene sequence. Strain JH145 could grow with quinoline as the sole carbon source. The optimum growth temperature, pH, and salt concentration were 30 °C, 8.0 and 1%, respectively, and 100 mg/L quinoline could be completely degraded within 28 h. Particularly, strain JH145 showed excellent quinoline biodegradation ability under a high-salt concentration of 7.5%. Two different quinoline degradation pathways, a typical 8-hydroxycoumarin pathway, and a unique anthranilate pathway were proposed based on the intermediates identified by HPLC–MS. Our present results provided new candidates for industrial application in quinoline-contaminated wastewater treatment even under high-salt conditions.
